# Supplementary material for: The concurrent impacts of drought and leaf harvesting on two traditional African vegetable non-timber forest product species
Source: PLoS One. 2023 Apr 5;18(4):e0283900. doi: 10.1371/journal.pone.0283900 (PMC10075472; doi:10.1371/journal.pone.0283900)
Supplement: Attachment — Submitted filename: Responses to reviewers.docx [file pone.0283900.s002.docx]

Corresponding Author: Gisele K. Sinasson S.

Department of Environmental Science

Rhodes University

Makhanda 6140, South Africa

Tel: +27 (0) 78 222 2100

Email: [sinasson.gisele@gmail.com](mailto:sinasson.gisele@gmail.com)

Dear Editor-in-Chief,

I’m pleased to submit a new version of the manuscript PONE-D-22-25341 entitled “**The concurrent impacts of drought and leaf harvesting on two traditional African vegetable non-timber forest product species”**.

Please, kindly find below the provided answers to the reviewers’ comments and suggestions.

We are very grateful for the reviewers’ efforts to improve the scientific quality of the manuscript by providing helpful comments and suggestions.

For clarity reasons, comments and suggestions of the reviewers (highlighted in grey) have been reported and responses provided accordingly below.

**Journal requirements**

1. Please ensure that your manuscript meets PLOS ONE's style requirements, including those for file naming.

The authors made all the necessary editing work to ensure that their manuscript meets the journal style

requirements.

2. Please remove any funding-related text from the manuscript and let us know how you would like to update your Funding Statement. Please include your amended statements within your cover letter; we will change the online submission form on your behalf.

The funding-related text was removed from the manuscript. The amended statement has been included in the new version of the cover letter.

3. Please complete your Competing Interests on the online submission form to state any Competing Interests. If you have no competing interests, please state ""The authors have declared that no competing interests exist."" This information should be included in your cover letter; we will change the online submission form on your behalf.

The information related to the Competing Interests has been included in the cover letter, as requested.

4. We note that you have stated that you will provide repository information for your data at acceptance. Should your manuscript be accepted for publication, we will hold it until you provide the relevant accession numbers or DOIs necessary to access your data. If you wish to make changes to your Data Availability statement, please describe these changes in your cover letter and we will update your Data Availability statement to reflect the information you provide.

Change to the Data Availability statement has been provided in the cover letter. The authors, hereby, confirm that the data related to the manuscript are now fully accessible through the provided DOI link.

**Reviewer 1**

***Methods***

A topsoil was used in the experiment. However, Information about the water tension of the soil under the drought treatment is missing. This information is important; otherwise it will be difficult repeating the experiment. I suggest adding this information, if available. Also, I suggest describing soil texture, as this trait affect water holding capacity. It is also missing the leaf water potential (or leaf water content) of plant subjected to water stress. If available, I suggest adding these data.

The authors thank the reviewer for the important comments. However, we were unable to collect data related to the water tension of the soil nor the leaf water potential during the experiment and so those data are not available.

As far as the soil texture is concerned, this information has been added to the methods section accordingly.

I suggest adding a figure describing the climatic conditions (light, relative humidity, temperature, and potential evapotranspiration) within the greenhouse during the experimental period.

The authors would have love to include such figure in the manuscript and for that some iButtons have been installed within the greenhouse during the experiment. However, due to some technical issues beyond our control, we were finally unable to obtain the climatic data.

L169: Information about the number of replications per treatment is missing. I suggest adding it.

The number of replications per treatment has been added for both species.

L141: … The maximum temperature in … ranged 18ºC-32ºC in
Suggestion: … the experiment, ranged from 18ºC to xx ºC in ..
(see others).

Change has been made in the sentences to consider the reviewer’s comment.

***Results***

L245: effect size of the drought … was slightly weaker for Amaranthus sp. than for B. pilosa
" was slightly weaker " ?
I suggest adding the p values after " was slightly weaker "
(see others)

The n value in Tables 2:
Table 2: The sample size (n value) is missing. I suggest adding the n for each species. See others (e.g. Table 3, 5, 6).

The required information has been added to the manuscript.

***Discussion***

On the negative growth (e.g. Table 3, 6).
I suggest commenting on this topic

The negative values of the growth have been commented in the discussion accordingly.

**Reviewer 2**

In the methodology, I suggest the author should include some pictures in order to attract the reader.

Pictures illustrating different phases of the experiment have been included, as suggested by the reviewer.

Tables should be re-drawn scientifically

The authors thank the reviewer for the comment but we would like to note that the tables have been designed following the journal guidelines. However, some modifications have been made for the tables to look a bit better.

L480 Where is the figure?

According to the journal guidelines, figures should not be included in the main manuscript file. Each figure must be prepared and submitted as an individual file. Figure captions must be inserted in the text of the manuscript, immediately following the paragraph in which the figure is first cited.

**All other suggestions, corrections and remarks by reviewer 2 for the improvement of the manuscript have been considered.**

I remain at your entire disposal, should you need any further information.

Kind regards,

Gisele K. Sinasson S.
